# Genesee Co. Medical Society

**Published:** 1870-01

**Authors:** L. L. Tozier

**Affiliations:** Secy.


### Genesee Co. Medical Society.

We have received, through the favor of Dr. Cotes, of Batavia, the following report of the proceedings of the Genesee County Medical Society, as published in the Batavia *Times:*

The semi-annual meeting of the Genesee Co. Medical Society was held at the St. James Hotel, in Batavia, Jan. 18, 1870.

The meeting was opened by the President, B. F. Benham, at 11 A. M. There was a good attendance of members for this season of the year, and everything passed off in a pleasant and harmonious manner.

Dr. John Root presented the name of E. W. Marsh, of Darien, who was elected to membership.

The following delegates were elected to represent this society at the next meeting of the New York State Central Association, which is to be held at Rochester in June next:

Drs. L. B. Cotes, J. Root, M. W. Townsend, F. W. Crane, and F. L. Stone.

The Society has recently adopted a new Constitution and form of By-Laws, and caused to have printed with them the Code of Ethics of the American Medical Association, which points out clearly the duty of physicians to their patients, to each other, and to the profession at large, and the duties of the profession to the public, and of the public to the Profession. Also a list of the names of the members of the Society since its organization in the year 1801.

Those of the public who may be interested in medical matters and wish to inform themselves in regard to the rules and regulations governing regularly authorized physicians, may obtain a copy by applying to any member of the Society.

After discussing at considerable length some points of interest only to the Society, L. B. Cotes introduced the following resolution, which was adopted.

*Whereas,* Infanticide, before birth is practised to an alarming degree in this country, and in the neighborhood of every physician, more or less, therefore—

*Resolved,* That the Genesee County Medical Society solemnly repudiates the practice, and that it is the duty of each member, as far as possible, to correct public sentiment upon this subject by laying it before his patrons at all suitable times, and to report any member guilty of practising this flagrant wickedness. And furthermore that the members of this Association consider themselves, and hereby form themselves into a vigilant committee to detect and report any member of the profession outside of the Society, or any charlatan. That they may confer with each other as to the best method to correct this physical and moral evil—at least in this county.

No more business appearing before the Society, it was adjourned.

L. L. TOZIER, *Secy.*

